# When Standard Is Not Enough: A Narrative Review of Supratherapeutic SSRI Doses in Resistant Obsessive Compulsive Disorder

**DOI:** 10.3390/jcm14113858

**Published:** 2025-05-30

**Authors:** Giacomo Gualtieri, Alessandro Cuomo, Simone Pardossi, Andrea Fagiolini

**Affiliations:** Department of Molecular Medicine, University of Siena School of Medicine, 53100 Siena, Italy; giacomo.gualtieri2@unisi.it (G.G.); alessandro.cuomo@unisi.it (A.C.); andrea.fagiolini@unisi.it (A.F.)

**Keywords:** obsessive–compulsive disorder, high-dose SSRIs, treatment resistance

## Abstract

**Background/Objectives**: OCD is a chronic psychiatric disorder, often requiring long-term pharmacological treatment. Although selective serotonin reuptake inhibitors (SSRIs) are considered first-line agents, 40 to 60% of patients show only partial or no response when treated at standard dosages. In such cases, supratherapeutic doses of SSRIs have been proposed as an alternative strategy. However, the evidence supporting this approach remains limited and fragmented. This review aims to evaluate the rationale, clinical efficacy, tolerability, and practical considerations associated with high-dose SSRI use in OCD. **Methods**: A structured narrative review was conducted using targeted literature searches in PubMed and Scopus. Studies were included if they reported on the use of high-dose SSRIs (citalopram, escitalopram, fluoxetine, fluvoxamine, paroxetine, or sertraline) in patients with OCD and provided efficacy and/or tolerability data. Clinical trials, observational studies, and case reports were all reviewed. **Results**: Evidence shows that higher doses of SSRIs are significantly more effective than low or medium doses in reducing OCD symptoms—especially in individuals who have only partially responded to standard treatment. Smaller clinical studies and case reports have also demonstrated that supratherapeutic dosing, beyond typical regulatory limits, can be both effective and well tolerated in treatment-resistant OCD. **Conclusions**: High-dose SSRI treatment may be a valuable option for selected OCD patients who do not respond to standard therapy. However, careful patient selection, regular monitoring, and further controlled studies are necessary to better define its long-term safety and effectiveness. In this context, increasingly advanced technologies—such as therapeutic drug monitoring and pharmacogenetic testing for relevant polymorphisms—may support more individualized and safer treatment strategies.

## 1. Introduction

Obsessive–compulsive disorder (OCD) is a common mental condition that affects around 2 to 3% of the population around the world and is often lifelong. It causes major disruption to life and diminished quality of living [1,2]. First-line pharmacological treatment mainly consists of selective serotonin reuptake inhibitors (SSRIs), which have been shown to be effective in lowering symptom severity by influencing the serotonergic neurotransmission [3,4]. However, around 40–60% of OCD patients do not respond adequately to standard SSRI dosages, which represents a major clinical problem given how debilitating treatment-resistant OCD (TR-OCD) is [5,6]. Such cases of TR-OCD are a challenging subgroup, often linked to serious and persistent mental illness, high comorbidity, and huge financial burdens due to the frequent use of medical services and the effects on the family [5,6]. Thus, several alternatives or adjunctive therapies have been employed for this cluster of patients. Combining pharmacological treatment with cognitive–behavioral therapy (CBT), especially exposure and response prevention (ERP), has been proven to be a widely adopted approach in clinical practice [7,8]. Another established therapeutic approach focuses on using atypical antipsychotic drugs to augment SSRIs, including risperidone, olanzapine, quetiapine, and aripiprazole. Such a combination might be effective when combined with additional neurotransmitters, such as dopamine, concurrent with serotonin. Several trials have demonstrated the efficacy of this kind of combination [9,10,11]. Changing to other serotonergic medications, such as clomipramine, a strong serotonin reuptake inhibitor, or using serotonin-norepinephrine reuptake inhibitors (SNRIs) like venlafaxine, can also alleviate symptoms, especially when comorbid depressive manifestations complicate the symptomatology [12,13]. Furthermore, new evidence supports the use of neuromodulation technologies, such as transcranial magnetic stimulation (TMS) and deep brain stimulation (DBS), particularly in cases of severe resistance [5].

The complex interplay between serotonin neurotransmission and dysfunctional cortico-striato-thalamo-cortical circuitry lies at the heart of OCD, as evidenced by the preferential effectiveness of SSRIs over other antidepressants [14]. Despite the therapeutic success of SSRIs, the specific serotonergic abnormalities underlying OCD remain not fully defined [15]. Some authors have hypothesized that SSRIs alleviate obsessive–compulsive symptoms by indirectly modulating hyperactive cortico-striato-thalamo-cortical (CSTC) circuits through serotonergic mechanisms, restoring balance between excitatory and inhibitory signaling within these neural networks [14].

Despite various therapeutic approaches, a substantial percentage of OCD patients—estimated at up to 20–30%—continue experiencing symptoms and functional impairments, highlighting the urgent need to understand and cultivate alternative individualized remedies [8,16]. Moreover, a recent study indicates that many OCD patients are undertreated before being classified as treatment-resistant [17]. In a large outpatient cohort, only 3% had received all guideline-recommended pharmacological steps, and fewer than one third had ever received SSRIs at the maximum dose [17].

Among emerging therapeutic strategies, clinicians occasionally consider administering high-dose SSRIs—dosages surpassing standard regulatory boundaries—as a potentially effective option in patients who exhibit partial but insufficient responses to standard administration [18]. This methodology is premised on the hypothesis that amplified serotonergic stimulation may be necessary in specific cases to achieve a sufficient modulation of persistent hyperactivity within cortico-striato-thalamo-cortical (CSTC) circuits, which are central to OCD pathophysiology [14]. Although observational and anecdotal evidence suggests possible benefits from this approach, rigorous clinical data remain limited, and issues pertaining to tolerability, side-effects, and safety underline the importance of careful risk–benefit assessments. The rationale, current evidence, clinical considerations, and safety implications of high-dose SSRIs constitute a critical area of ongoing investigation, and these aspects will be examined comprehensively in subsequent sections of this review. Specifically, we aimed to explore whether supratherapeutic doses of SSRIs provide a clinically meaningful improvement in TR-OCD and whether such benefits outweigh the associated risks.

## 2. Materials and Methods

This review explores the clinical rationale, therapeutic potential, safety considerations, and current evidence supporting the use of high-dose SSRIs in TR-OCD, aiming to guide clinicians in making informed decisions when confronted with partial or non-responders.

This work was conducted as a narrative review with a structured literature search, aiming to summarize the available clinical evidence on the use of supratherapeutic doses of SSRIs in the treatment of TR-OCD.

### 2.1. Literature Search Strategy

A targeted electronic search was performed in PubMed and Scopus in March 2024 using the following Boolean search string:

((citalopram OR sertraline OR paroxetine OR escitalopram OR fluvoxamine OR fluoxetine) AND obsessive) AND (supratherapeutic OR high-dose OR high dose).

No date restrictions were applied. Only articles published in English and involving human subjects were considered.

### 2.2. Study Selection

After duplicate removal, two independent reviewers (GG and SP) screened all titles and abstracts for relevance. Articles published between January 1990 and March 2025 were considered. Only articles published in English and involving human subjects were included, regardless of the country of origin. Full-text articles were then assessed according to the following inclusion criteria:

− Clinical studies (randomized controlled trials, open-label studies, observational studies, or case series);
− Reporting on the use of high-dose SSRIs in patients with a primary diagnosis of obsessive–compulsive disorder;
− Providing data on efficacy and/or tolerability outcomes.

Exclusion criteria included the following:

− Studies focused on other psychiatric conditions or comorbidities;
− Pharmacokinetic or animal studies;
− Articles not providing original clinical data (e.g., editorials, letters, reviews without patient data).

Disagreements between reviewers were resolved through discussion and, if needed, a third senior reviewer (AC).

### 2.3. Data Extraction and Synthesis

For each included study, the following data were extracted independently by the same two reviewers:

− Study design and sample size;
− Type and dosage of SSRI used;
− Outcome measures for efficacy and side-effects;
− Main findings and conclusions.

The analysis focused on four dimensions, identified a priori based on clinical relevance and literature gaps: efficacy data, safety/tolerability, and clinical considerations.

Due to the heterogeneity of study designs and outcome metrics, a narrative synthesis was performed. No quantitative meta-analysis was conducted.

## 3. Rationale for High-Dose SSRI Use in OCD

The primary pharmacological rationale for using supratherapeutic SSRI doses in treating OCD is related to the pathophysiological mechanism underlying OCD. Particularly, SSRIs achieve their therapeutic effects by selectively inhibiting serotonin (5-HT) reuptake transporters, thereby heightening serotonergic neurotransmission [19]. OCD patients are hypothesized to require significantly higher synaptic serotonin levels compared to patients with depression or anxiety disorders, potentially due to differences in serotonergic neuron sensitivity or receptor functionality [6]. SSRI dosages used to manage depressive symptoms usually result in a serotonin transporter (SERT) occupancy of about 70–80% [20]. In contrast, OCD symptoms might require a higher degree of SERT inhibition, potentially over 80% [6,21].

Moreover, individual differences in SSRI pharmacokinetics can occur. For instance, variations in hepatic metabolism (such as cytochrome P450 polymorphisms) can influence drug plasma levels and thus its CNS bioavailability [22]. Patients identified as rapid metabolizers may present lower drug plasma concentrations at standard therapeutic doses, hence requiring higher doses to achieve therapeutic serum levels and adequate pharmacological effects [22]. Consequently, supratherapeutic dosing can be pharmacokinetically justified to overcome individual metabolic variations and ensure optimal central nervous system drug levels [22].

Additionally, recent evidence suggests that genetic factors may contribute to SSRI non-response, particularly in patients with anxiety and depressive disorders. A 2025 study by Hanna et al. found that individuals carrying both the SLC6A4 short/short (SS) genotype and the HTR1A rs6295 G/G genotype were significantly overrepresented among those who failed to respond to standard-dose SSRIs [23]. This genetic combination appears to reduce serotonin transporter availability and impair autoreceptor desensitization, limiting the therapeutic effects of SSRIs. Further studies have identified additional gene variants—such as SLC6A4 rs16965628, COMT rs4680, and GRIN2B rs1019385—that may influence SSRI response through effects on serotonergic tone and synaptic plasticity [24]. In such cases, supratherapeutic dosing may help overcome reduced pharmacodynamic sensitivity.

Finally, long-term SSRI treatment induces adaptive neuroplastic changes including the downregulation of postsynaptic 5-HT receptors and changes in intracellular signaling cascades, possibly critical for their therapeutic mechanisms in obsessive–compulsive disorder [25]. Higher doses of SSRIs may speed up or amplify these adaptive neurochemical and neuroplastic mechanisms and thus provide better control of TR-OCD symptoms.

## 4. Clinical Evidence for High Dosing

Considering the recommended SSRI dosages for OCD, clinical evidence strongly supports the use of high doses, particularly among individuals who exhibit an inadequate response to standard therapeutic doses [6,26].

A meta-analysis including over two thousand patients showed that higher doses of SSRIs were significantly superior to both medium and low doses in reducing OCD symptom severity, as measured by changes in the Yale–Brown Obsessive–Compulsive Scale (Y-BOCS) [6]. The weighted mean difference between high-dose SSRIs and placebo was −3.9 points, and statistically significant superiority was also observed when high doses were compared to medium or low ones [6]. The clinical response rate—a decrease of 25% or 35% in Y-BOCS score—was more likely to be achieved at higher doses, with an estimated number needed to treat (NNT) of 4.5 compared to placebo, and approximately 12.5 when high versus medium doses were compared [6]. Considering the tolerability profile, although side-effects are more frequent at higher doses, this typically results in only a slight increase in treatment discontinuations due to adverse events, with a number needed to harm (NNH) of approximately 14 when comparing high doses to placebo [6]. Importantly, the overall acceptability of treatment, as assessed by all-cause dropout rates, did not significantly differ across dose levels [6].

Further confirmation of these findings comes from a dose–response meta-analysis that utilized fluoxetine-equivalent conversions to standardize doses across a broad range of SSRIs, thereby increasing the generalizability of the data [26]. This analysis revealed a monotonic increase in efficacy up to approximately 40 mg/day of fluoxetine equivalents, after which the therapeutic benefit plateaued. Notably, the curve representing dropout due to adverse effects rose steadily with increasing dose, while the curve for all-cause dropout remained flat [26]. This reinforces the idea that although high doses are associated with increased side-effect burden, overall treatment continuation is not compromised [26].

## 5. Supratherapeutic SSRI Doses in Resistant OCD

Some studies have specifically considered the use of supratherapeutic SSRI doses to treat OCD (Table 1).

For instance, a multicenter, double-blind randomized controlled trial [27] was conducted to evaluate the efficacy and tolerability of high-dose sertraline in OCD patients who had not responded to 16 weeks of standard-dose treatment (up to 200 mg/day). Sixty-six non-responders were randomized to receive either a flexible high dose ranging from 250 to 400 mg/day or to continue on a fixed dose of 200 mg/day for an additional 12 weeks. The results indicated that the high-dose group experienced a significantly greater and more rapid symptom improvement across multiple measures. In particular, a faster reduction was observed in YBOCS scores, the National Institute of Mental Health-Global Obsessive–Compulsive Scale (NIMH-GOCS), and Clinical Global Impressions (CGIs). Although the overall response rates at the endpoint (defined as a ≥25% reduction in YBOCS score and a CGI-I score ≤ 3) did not differ significantly between groups—40% in the high-dose group versus 33% in the 200 mg/day group—a higher proportion of completers in the high-dose group met the response criteria (52% vs. 34%), suggesting a potential clinical benefit from extended treatment at elevated doses. Both dosing regimens were similarly well tolerated. Adverse event rates did not significantly differ between groups, and no patients in the high-dose group discontinued due to side-effects. Common adverse events such as insomnia, diarrhea, nausea, and somnolence occurred less frequently than previously reported in standard-dose trials [27].

To determine the efficacy and tolerability of supratherapeutic doses of escitalopram over 16 weeks in OCD patients, an open-label randomized trial [28] comparing 20 mg/day and 30 mg/day was conducted. Thirty subjects were enrolled and randomized to one of the two dosing regimens following initial titration. Across both treatment groups, statistically significant improvements were observed in all clinical measures. Post hoc analyses indicated that both treatment groups experienced significant improvement in OCD symptoms, anxiety, depression, and quality of life. Among study completers (n = 23), 52.2% met the full response criteria. Specifically, in the 30 mg/day group, 15 out of 20 completers (75%) achieved a full response, compared to 8 out of 13 (61%) in the 20 mg/day group. While these between-group differences did not reach statistical significance after adjusting for baseline differences in mood and anxiety, patients in the higher-dose group demonstrated greater mean reductions in Y-BOCS scores and better outcomes across multiple secondary measures. The effect size for Y-BOCS improvement was large (Cohen’s d = 0.79), supporting the possibility of a dose–response relationship. Adverse events were generally mild to moderate and comparable between the two groups. These findings suggest that 30 mg/day of escitalopram may be more effective than 20 mg/day in patients with comorbid affective symptoms, although further controlled studies are necessary to confirm this advantage [28].

A 16-week open-label prospective study [29] evaluated the efficacy and tolerability of high-dose escitalopram (up to 50 mg/day) in patients with OCD who had failed to respond after four weeks of standard-dose treatment (20 mg/day). Sixty-seven individuals with severe OCD were enrolled. Following the initial phase, only two patients met the response criteria (≥25% reduction in Y-BOCS) and continued on 20 mg/day, while the remaining 64 were titrated to a mean dose of 33.8 mg/day by the study endpoint. At week 16, substantial improvements were observed across all clinical measures, including obsessive–compulsive symptoms, depressive symptoms, overall severity, functioning, and global improvement. Over 80% of patients treated with high doses achieved a full response (≥25% reduction in Y-BOCS). The treatment was well tolerated; no patients discontinued therapy due to adverse effects. The most commonly reported adverse events were decreased sexual desire (31.8%)—primarily in males—and dry mouth (12.1%). One patient developed hypomania at 45 mg/day, which resolved after the dose was reduced [29].

Another study [30] on escitalopram evaluated, over a 12-week period using an open-label pilot design, the effectiveness and tolerability of a supratherapeutic dose of 30 mg/day in 11 adult outpatients with OCD. Scitalopram was initiated at 10 mg/day and titrated to 30 mg/day by week 4, which was maintained for the remainder of the study. At the endpoint, 54.5% of patients achieved a ≥40% reduction in Y-BOCS scores, and 81.8% were classified as responders when using a 25% reduction threshold. The mean total Y-BOCS score decreased by 39.6%, with even greater reductions observed in the obsession and compulsion subscales. Treatment was well tolerated: only two patients reported mild adverse effects (decreased libido, excessive sweating, and delayed ejaculation), and no participants discontinued due to side-effects [30].

Two case reports [31,32] illustrate that supratherapeutic doses of SSRIs may provide meaningful clinical benefits in treatment-resistant OCD. In one case [32], three different pharmacological strategies had failed, yet the patient experienced significant and sustained improvement only after citalopram was increased to 160 mg/day. In another report [31], a previously unresponsive patient showed marked symptom improvement, including a reduction in Y-BOCS score, after sertraline was increased from 200 mg/day to 300 mg/day. These cases suggest that very high-dose SSRI strategies may have a potential role in highly individualized treatment approaches for resistant OCD. However, they also emphasize the critical importance of safety monitoring, particularly in relation to cardiovascular risk, neurological side-effects, and the potential for serotonin toxicity when exceeding standard dosage recommendations.

## 6. Clinical Risks: Balancing Risks and Benefits

In a few studies, the treatment of OCD with the use of supratherapeutic doses of SSRIs has shown clinical utility—particularly in treatment-resistant cases—but it is also accompanied by increased adverse effects that are directly proportional to the dose required for efficacy [6]. In a meta-analysis of nine randomized controlled trials, high-dose SSRIs showed clearer evidence of superiority compared to low and medium doses; at the same time, however, they caused a greater number of dropouts due to side-effects [6].

Particularly worrisome side-effects of high-dosage SSRIs include QTc interval prolongation, especially noted with citalopram and escitalopram [33,34]. This form of cardiac toxicity is dose-dependent and can culminate in torsades de pointes, particularly in patients with predisposing factors such as electrolyte imbalances or those taking concomitant QT-prolonging medications. Regulatory agencies have advised that, because of this risk, the citalopram dosage should not exceed 40 mg [33,34]. Another potentially lethal complication of high-dose SSRI use is serotonin syndrome, which results from excessive serotonergic activity. Although rare, cases have been reported even with monotherapy, as well as in combination with other serotonergic drugs such as MAOIs or triptans (Edinoff et al., 2021) [33,34]. Sexual dysfunction—which includes reduced libido, delayed ejaculation, and anorgasmia—is both common and dose-dependent, and, at higher doses, can affect up to 75% of patients, particularly those taking paroxetine and citalopram [33,34]. Gastrointestinal side-effects—including nausea, diarrhea, and abdominal discomfort—are also common obstacles to dose escalation. These symptoms are particularly frequent with fluvoxamine and sertraline [33,34]. Hyponatremia is another hazard, especially in older adults or those taking diuretics; it is usually due to the syndrome of inappropriate antidiuretic hormone secretion (SIADH), and has been observed most frequently with fluoxetine and paroxetine [33,34].

Weight gain, though variable across different agents, is a well-documented effect with chronic SSRI use. The most significant gains are seen with paroxetine and sertraline, with reports of increases up to 10 kg over the course of a year [33,34]. Extrapyramidal symptoms (EPSs)—most notably tremor, but also chorea and parkinsonism—although rare, have been reported in association with high-dose SSRI use, possibly due to the serotonergic modulation of dopaminergic pathways [33,34].

However, a study specifically considered the safety and tolerability of supratherapeutic doses of SSRIs for OCD [35]. A total of 105 outpatients aged 18 or older, diagnosed with OCD, were treated for more than six months with supratherapeutic doses of SRIs—specifically, sertraline > 200 mg/day (up to 650 mg), escitalopram > 20 mg/day (up to 80 mg), fluoxetine > 60 mg/day (up to 120 mg), and fluvoxamine > 300 mg/day (up to 600 mg). The results demonstrated that high-dose SSRIs were generally well tolerated, with no reported cases of serotonin syndrome or DILI. The most commonly reported side-effects were sexual dysfunction (34%), weight gain (27%), and sedation (26%). Hyperhidrosis occurred in 19% of participants, while tremor was reported in 10%. Notably, only one case of QT interval prolongation (QTc = 580 ms) and one case of first-time seizure were observed; both adverse events were self-limiting and did not recur. Crucially, the overall rate of side-effects did not differ significantly among the three dosing groups (≤200 mg, 201–400 mg, and 401–650 mg sertraline-equivalent doses), suggesting that tolerability was not dose-dependent within the tested range [35].

## 7. Clinical Practice Considerations

The findings from this review may suggest that, in selected TR-OCD patients, above-therapeutic doses of SSRIs could be efficacious and well tolerated. This consideration becomes particularly relevant in light of a recent systematic review of approved psychiatric medications, which emphasized that no new pharmacological treatments for OCD have been approved in recent years [36], thereby reinforcing the clinical need to optimize existing options, such as high-dose strategies, when standard interventions prove insufficient. However, the heterogeneity of trials highlights the importance of individually assessing risks and benefits. For example, while the Ninan et al. study [27] found significant improvement with sertraline doses above 250 mg/day, the response rate did not differ significantly at the endpoint compared to the standard dose. Conversely, Rabinowitz et al. [29] reported robust response rates (>80%) with escitalopram up to 50 mg/day, suggesting drug-specific dose–response profiles. A striking gap across the reviewed studies is the lack of long-term follow-up data. Most trials lasted 12 to 16 weeks, which limits our ability to draw conclusions about sustained efficacy, tolerability, and metabolic or cardiovascular effects of high-dose SSRI therapy. Before administering supratherapeutic doses of SSRIs to patients suffering from OCD, several clinical checks must be performed to maximize efficacy and minimize harm. It is necessary for the clinician to verify that the patient has adhered to the prescribed dosage and has taken the medication for an adequate duration before concluding that the patient is a non-responder [37].

A comprehensive review of medical and psychiatric comorbidities is essential, especially conditions that may increase vulnerability to side-effects, such as cardiac arrhythmias, epilepsy, or hyponatremia in the elderly [38]. Particular attention should be paid to drug–drug interactions, given that many SSRIs inhibit hepatic cytochrome P450 enzymes (notably CYP2D6 and CYP3A4) [39]. These interactions—especially those leading to increased serum concentrations of co-administered drugs—can reduce the therapeutic window and offset potential benefits [39].

Several genetic polymorphisms affecting SSRI metabolism have been identified, such as CYP2C19-poor metabolizer status for citalopram and escitalopram, which can lead to elevated plasma levels and a greater risk of toxicity, even at standard doses [40]. As previously discussed, pharmacodynamic variants—such as the 5-HTTLPR S/S genotype in the SLC6A4 gene and the rs6295 G/G variant in the HTR1A gene—may also play a role in limiting SSRI efficacy and should be considered when evaluating treatment resistance [23,24]. Ideally, given the potential influence of genetic polymorphisms on SSRI response, genetic testing that investigates such variants could become increasingly relevant in guiding therapeutic choices and dosing strategies.

Moreover, therapeutic drug monitoring (TDM) may represent a valuable tool to support the safe implementation of high-dose SSRI strategies [41]. By measuring plasma concentrations under steady-state conditions, TDM enables clinicians to individualize treatment regimens based on pharmacokinetic variability, detect subtherapeutic or toxic levels early, and improve adherence through shared decision making [41]. Recent technological advances—such as dried blood spots and saliva-based assays—may facilitate the broader clinical adoption of TDM in psychiatry, even in non-hospital settings [41].

Once treatment has commenced, structured clinical monitoring should be ongoing. This may include ECG monitoring for QTc intervals in patients taking citalopram, escitalopram, or those with cardiac risk factors [42]. Side-effects in gastrointestinal, sexual, and neuropsychiatric domains should be proactively assessed and documented in routine clinical interviews, as these are both common and likely to affect treatment adherence [42].

Perhaps most importantly, the clinician should have a plan in place for discontinuation in the event of inefficacy or intolerable side-effects. SSRIs administered at high doses should be tapered gradually to minimize withdrawal symptoms. The pharmacokinetics of certain agents—such as paroxetine and venlafaxine—may necessitate additional care due to their shorter half-lives [43].

## 8. Limitations

A major limitation of this review lies in its narrative nature, as it was not systematically designed. A systematic review was not pursued, due to the limited number of available studies on supratherapeutic SSRI dosing in OCD, none of which included large sample sizes. Moreover, the scarcity of randomized controlled trials—many of which are also relatively dated—along with heterogeneity in study designs, dosing strategies, outcome measures, and follow-up durations, would have limited the feasibility and meaningfulness of a formal meta-analytic synthesis. Nevertheless, we followed a structured search and selection process and attempted to present the clinical evidence as rigorously as possible within the constraints of a narrative format. From a clinical point of view, very few studies have evaluated the long-term safety profile of supratherapeutic SSRI doses beyond 16–24 weeks, while in clinical practice, such regimens may be prolonged, especially in severe TR-OCD cases.

## 9. Conclusions and Future Directions

High-dose SSRI strategies should be considered primarily in patients with a clearly documented partial response to standard treatment, after confirming adherence and excluding contraindications. Routine ECG monitoring, particularly for citalopram and escitalopram, is warranted, and educating patients about potential side-effects remains critically important. Furthermore, the consistent absence of serotonin syndrome or serious cardiovascular events in the reviewed trials supports the case for real-world studies assessing long-term safety in naturalistic settings. More well-designed, large-scale randomized controlled trials are necessary to compare the high-dose antidepressant strategy with standard-dose SSRI therapy in patients with TR-OCD—especially those who have only partially responded or not responded at all to traditional regimens.

Future research should place greater emphasis on therapeutic drug monitoring. Measuring plasma SSRI levels allows for individualized dose adjustments and can be particularly useful in cases of suspected rapid metabolism or subtherapeutic exposure, even when the patient is adherent to the treatment. With the growing availability and accuracy of TDM tools—which were previously unavailable or less precise—clinicians may now rely on these methods to safely administer high doses of SSRIs [41,44]. In parallel, growing evidence supports the value of genetic testing—particularly for well-characterized polymorphisms in the cytochrome P450 system (such as CYP2C19 and CYP2D6)—that directly influence SSRI metabolism. Personalizing dosing based on metabolic profiles may lead to better outcomes while reducing the burden of side-effects. Despite growing clinical interest, the literature on supratherapeutic SSRI dosing in OCD remains sparse and mostly outdated. This highlights the importance of further investigation into a strategy that may offer significant benefits in treatment-resistant cases. Finally, more studies are needed to determine how high-dose strategies hold up over time. We need prospective data on their long-term safety and sustainability, including possible effects on metabolic health, cardiovascular function, and neuropsychiatric stability across extended treatment periods.

## Figures and Tables

**Table 1:** Key studies on high-dose SSRI use in OCD.

Study	Drug	Dose Range	Study Type	Sample Size	Key Findings
Ninan et al., 2004 [27]	Sertraline	200 vs. 250–400 mg/day	RCT	66	Higher response rate among completers in the 250–400 mg/day group, despite no significant difference at endpoint
Dougherty et al., 2009 [28]	Escitalopram	20 vs. 30 mg/day	Open-label	30	Higher response in the 30 mg/day group with large effect size, though not statistically significant after baseline adjustment
Rabinowitz et al., 2008 [29]	Escitalopram	Up to 50 mg/day	Open-label	67	≥80% responded at mean dose of 33.8 mg/day; significant improvement in OCD symptoms, depressive symptoms, global severity, functioning, and overall clinical impression
Galvão-de Almeida et al., 2007 [30]	Escitalopram	30 mg	Open-label	11	81.8% of patients achieving ≥25% improvement and good tolerability; no discontinuations occurred
Byerly et al., 1996 [31]	Sertraline	300 mg/day	Case report	1	Marked symptom improvement
Bejerot et al., 1998 [32]	Citalopram	160 mg/day	Case report	1	Marked symptom improvement
